# Fatal Suppurative Bronchopneumonia Associated With Multidrug‐Resistant *Klebsiella pneumoniae* in a Pet Rat (*Rattus norvegicus*): A Case Report

**DOI:** 10.1155/crve/3798536

**Published:** 2026-11-04

**Authors:** Jéssica Siqueira Sandrini, Gustavo Euclides de Oliveira Nascimento, Bianca Gianola Belline Silva, Kessie Marie de Moura Crescêncio, Marco Vinícius Chaud

**Affiliations:** ^1^ University of Sorocaba Veterinary Medicine Sorocaba São Paulo Brazil uniso.br; ^2^ University of Sorocaba Laboratory of Biomaterials and Nanotechnology (LaBNUS) Sorocaba São Paulo Brazil uniso.br

**Keywords:** Enterobacterales, *Klebsiella pneumoniae*, multidrug-resistant bacteria, rodent diseases, zoonoses

## Abstract

Respiratory diseases are common in companion rodents, but empirical treatment without microbiological diagnosis remains widespread, contributing to treatment failures and the selection of resistant strains. This study reports a case of fatal suppurative bronchopneumonia associated with a multidrug‐resistant (MDR) *Klebsiella pneumoniae* isolate in a pet rat (*Rattus norvegicus*). Following clinical presentation, serial thoracic radiographs were obtained, and empirical antimicrobial and respiratory therapies were administered. Because of progressive disease, the animal was humanely euthanized. Necropsy revealed multiple pulmonary abscesses, hemorrhage, and emphysema, with histopathology consistent with severe neutrophilic bronchopneumonia. *K. pneumoniae* was isolated from lung tissue in pure culture. Antimicrobial susceptibility testing according to BrCAST guidelines demonstrated resistance to doxycycline, chloramphenicol, enrofloxacin, marbofloxacin, and norfloxacin; intermediate susceptibility to cefazolin, streptomycin, gentamicin, and trimethoprim–sulfamethoxazole; and susceptibility to amikacin, aztreonam, ceftriaxone, cefotaxime, ceftazidime, and cefepime. This resistance profile provides a plausible explanation for the failure of empirical therapies with doxycycline and fluoroquinolones, although advanced pulmonary abscessation and lack of antemortem testing also influenced the outcome. The absence of molecular characterization and environmental screening limits definitive transmission inferences. Early microbiological investigation is essential to guide effective therapy, support antimicrobial stewardship, and mitigate potential public health risks at the human–animal interface within the One Health framework.

## 1. Introduction

Respiratory tract infections represent one of the most frequent clinical challenges in companion rodent medicine, particularly in pet rats (*Rattus norvegicus*) [1]. Because diagnostic investigations in small exotic mammals are often limited by patient instability, small anatomical size, or technical constraints, antimicrobial therapies are frequently initiated on an empirical basis [1, 2]. This practice can lead to treatment failure and contributes significantly to the selective pressure driving antimicrobial resistance (AMR) at the human–animal–environment interface within the One Health framework [2, 3].

*Klebsiella pneumoniae* is a Gram‐negative, facultative anaerobic enterobacterium recognized as an opportunistic pathogen capable of causing severe respiratory, urinary, and systemic infections in compromised hosts [4]. Recent genomic and epidemiological surveillance studies have increasingly documented multidrug‐resistant (MDR) and extended‐spectrum *β*‐lactamase (ESBL)‐producing *K. pneumoniae* lineages across diverse animal reservoirs, including livestock, poultry, wildlife, and food animals [5–7]. Although *K. pneumoniae* is well established in human nosocomial environments and production animals, reports documenting spontaneous, fatal clinical disease associated with MDR strains in pet rodents remain scarce [3, 8, 9]. Given the close physical proximity, direct handling, and shared indoor living spaces between companion rodents and their owners, the emergence of MDR enterobacteria in pet species highlights their potential role as sentinels or indirect indicators for critical resistance determinants circulating in the domestic environment [3, 8, 9]. To the authors′ knowledge, this report represents the first documented case of fatal suppurative bronchopneumonia associated with an MDR *K. pneumoniae* isolate in a pet rat (*R. norvegicus*) in Brazil. This report is aimed at describing clinical progression, serial radiographic findings, postmortem pathological characteristics, bacterial isolation, and antimicrobial susceptibility profile of this case, while critically discussing the therapeutic challenges, study limitations, and potential public health implications.

## 2. Case Report

An intact female pet rat (*R. norvegicus*), approximately 2 years old, weighing 258 g (measured at initial presentation, D0), was presented to a veterinary clinic with severe dyspnea, respiratory noise, prostration, and progressive weight loss. The animal had been owned since birth, was housed in a cage providing approximately 3 cubic feet (0.085 m^3^) per individual, with daily cleaned hay bedding, and was fed a commercial extruded rodent diet [1, 2]. The rat had no prior record of illnesses, no known previous antimicrobial exposure, and was housed exclusively with female cage mates.

Physical examination on Day 0 (D0) revealed a body condition score (BCS) of 2/5, hyperthermia at 40°C (physiological reference range: 37.5°C–38.5°C), orthopneic positioning, marked ocular secretion, and porphyrin nasal discharge (chromodacryorrhea) [10]. Thoracic auscultation demonstrated bilateral moist crackles. Because of acute respiratory distress, oxygen therapy was initiated in an oxygen chamber. Serial thoracic radiographs were obtained without chemical sedation, utilizing standard positioning and identical exposure settings across all evaluation points. The initial projections (D0) demonstrated diffuse radiopacity in the cranial thoracic cavity and focal pulmonary opacification (Figure 1).

**Figure 1:**
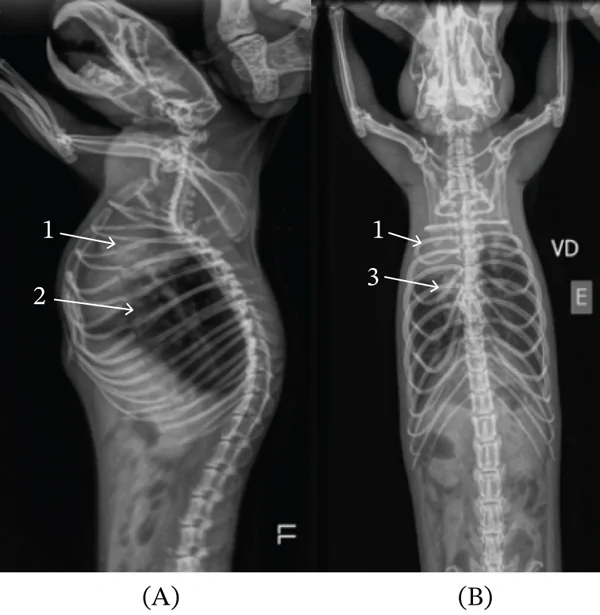
Thoracic radiographs of a pet rat (*Rattus norvegicus*) on Day 0 (D0). (A) Left lateral and (B) ventrodorsally thoracic projections obtained without chemical sedation on Day 0 (D0). Note the diffuse radiopacity in the cranial thoracic cavity and focal pulmonary opacification with partial obliteration of the cardiac silhouette.

Based on clinical presentation and common endemic respiratory pathogens in companion rodents, presumptively targeting murine respiratory mycoplasmosis, an empirical oral protocol was initiated consisting of doxycycline (5 mg/kg PO BID for 21 days) and systemic prednisolone (1 mg/kg PO BID for 5 days) [11].

On Day 10 (D10), slight clinical improvement was noted, though exercise intolerance and nasal discharge persisted. On Day 12 (D12), repeat thoracic radiography in left lateral and ventrodorsally projections revealed progressive homogeneous consolidation in the right cranial hemithorax with dorsal tracheal deviation, highly suggestive of pulmonary abscessation or neoplasia (Figure 2). Inhaled beclomethasone dipropionate (2 mg/kg BID for 5 days, then q48 h) was added via a modified pediatric spacer attached to a mask to mitigate local airway inflammation [10, 11].

**Figure 2:**
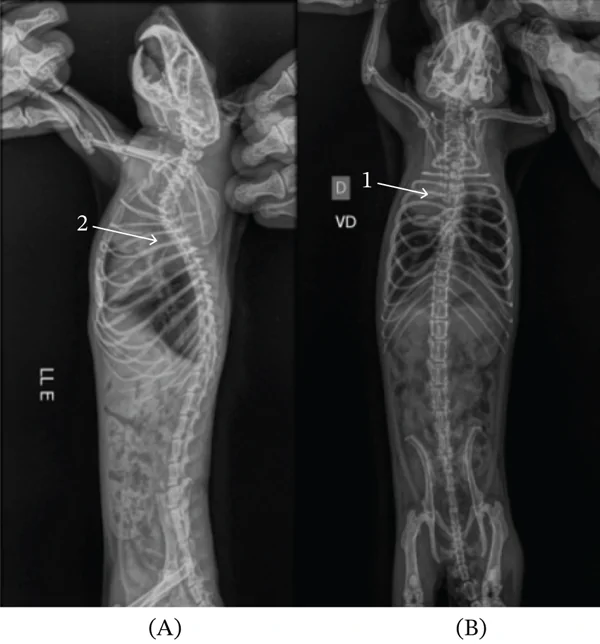
Thoracic radiographs of a pet rat (*Rattus norvegicus*) on Day 12 (D12). (A) Follow‐up left lateral and (B) ventrodorsal thoracic projections on day 12 (D12). Progressive homogeneous consolidation is visible in the right cranial hemithorax, associated with dorsal deviation of the thoracic trachea, highly suggestive of pulmonary abscessation or neoplasia.

On Day 17 (D17), persistent respiratory noise remained, although appetite improved. Inhalation therapy was adjusted to a combination of salmeterol xinafoate and fluticasone propionate (25 *μ*g/125 *μ*g, BID for 5 days, then SID for 10 days) using the modified spacer [4, 11].

On Day 32 (D32), the patient experienced severe clinical deterioration, presenting severe dyspnea, cyanotic mucous membranes, hypoxemia, and secondary neurological signs. Emergency radiography confirmed extensive cranial thoracic consolidation, diffuse multifocal pulmonary opacities, severe tracheal displacement, and secondary gastrointestinal aerophagia (Figure 3).

**Figure 3:**
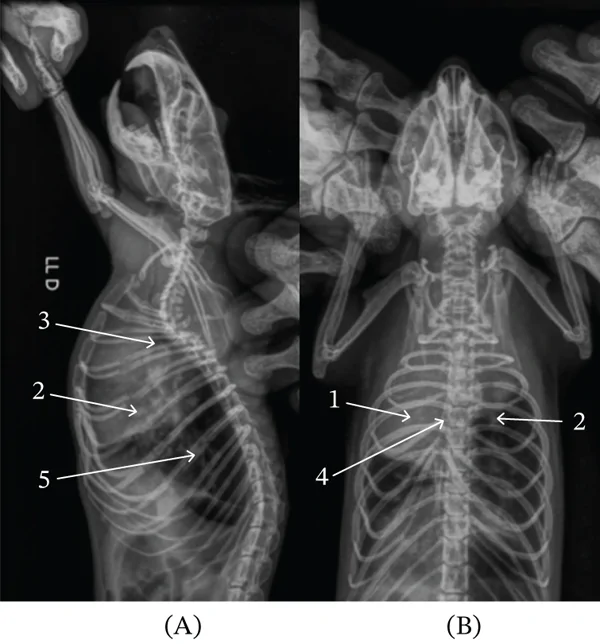
Thoracic radiographs of a pet rat (*Rattus norvegicus*) on Day 32 (D32). (A) Right lateral and (B) ventrodorsal thoracic projections on Day 32 (D32) during terminal decompensation. Marked cranial thoracic consolidation, diffuse multifocal pulmonary opacities, severe dorsal displacement of the trachea, loss of cardiac silhouette definition, and secondary gastrointestinal aerophagia are demonstrated.

Emergency administration of enrofloxacin 2.5% (2.5 mg/kg IM) was performed [11]. Additionally, oral itraconazole suspension (2.5 mg/kg PO) was administered empirically because of nonresponsive chronic pulmonary consolidation and clinical suspicion of opportunistic fungal infection [11]. Given the grave prognosis and lack of response to therapy, humane euthanasia was elected following full written owner consent [1, 12]. Euthanasia was elected because of grave prognosis and terminal respiratory failure. Initial inhalational anesthetic induction with isoflurane (5% in 100% O_2_) failed because of severe reduction in functional tidal volume. Anesthesia was subsequently induced via intramuscular injection of ketamine (100 mg/kg) and xylazine (10 mg/kg). Complete loss of consciousness and surgical anesthetic depth were rigorously confirmed by the complete absence of pedal withdrawal, palpebral, and corneal reflexes, alongside full muscular flaccidity. Following anesthetic verification, intraperitoneal thiopental sodium (90 mg/kg) was administered. Intracardiac administration of 10% potassium chloride (2 mEq/kg) was performed solely after confirming profound unconsciousness, in strict compliance of the AVMA guidelines. Irreversible cardiac arrest was confirmed by continuous thoracic auscultation [10–12].

The overall chronological management, clinical findings, diagnostic procedures, and therapeutic interventions are summarized in Table 1 according to CARE reporting guidelines.

**Table 1:** Chronological management, clinical findings, diagnostic procedures, and therapeutic interventions.

Day	Clinical findings	Diagnostics	Treatment	Outcome
D0	Dyspnea, hyperthermia (40°C), chromodacryorrhea, crackles, BCS 2/5	Serial radiography without sedation	Oxygen therapy, doxycycline (5 mg/kg PO BID), prednisolone (1 mg/kg PO BID)	Temporary stabilization
D10	Exercise intolerance, persistent nasal discharge	Repeat thoracic radiography: consolidation or Abscessation	Doxycycline	Persistent disease progression
			Inhaled	
D12	Pulmonary consolidation		Beclomethasone	
D17	Respiratory noise, appetite improvement light	Revaluation	Inhalation	Partial symptomatic relief
			Salmeterol and	
			Fluticasone	
D32	Severe respiratory distress	Thoracic radiography: signals consolidation and aerophagia	Enrofloxacin (2.5 mg/kg IM), Itraconazole (2.5 mg/kg PO)	Unfavorable evolution; humane euthanasia
	Cyanosis			
	Neurological signs			
Postmortem	Multiple pulmonary abscesses	Histopathology, bacterial culture, biochemical profiling, antibiogram diffusion′s disk	N/A	Isolation of MDR *K. pneumoniae*
	Suppurative bronchopneumonia			

Postmortem examination revealed severe pathological alterations restricted to the thoracic cavity, with no gross extrapulmonary or systemic lesions observed upon complete systemic inspection (Figure 4). Systemic dissection of the respiratory tract demonstrated widespread pulmonary involvement characterized by dark red consolidated areas, multifocal parenchymal hemorrhage, severe subpleural emphysema, and multiple parenchymal abscesses containing purulent exudate. Two large, encapsulated abscesses, measuring approximately 1.0 × 0.8 cm and 0.7 × 0.5 cm, were localized within the right cranial lung lobe and the cranial portion of the left cranial lung lobe, respectively [13].

**Figure 4:**
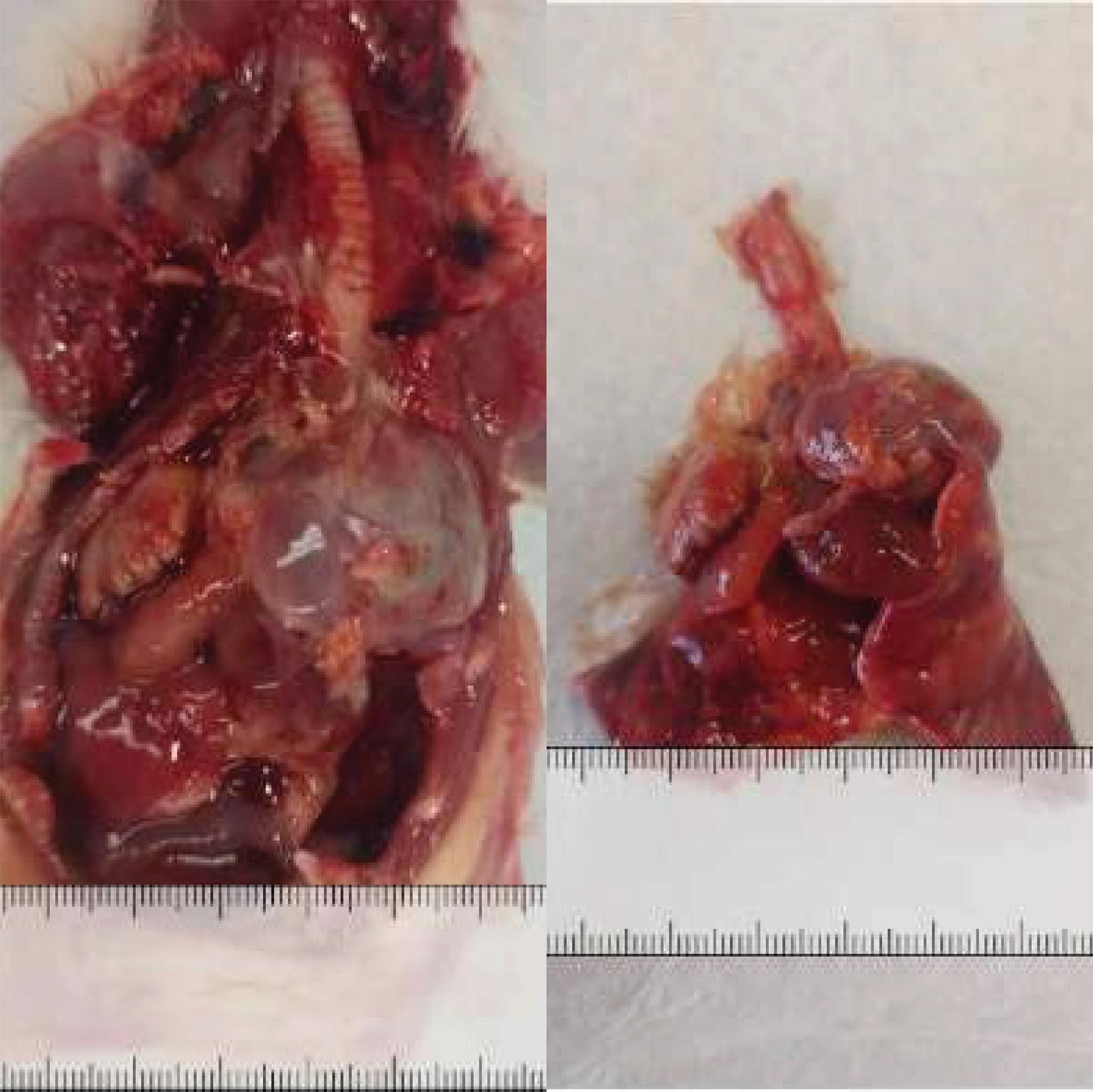
Gross inspection of the thoracic cavity shows severe, diffuse pulmonary consolidation, multifocal parenchymal hemorrhage, subpleural emphysema, and multiple purulent abscesses.

Gross inspection of the thoracic cavity displaying severe, diffuse pulmonary consolidation, multifocal areas of parenchymal hemorrhage, subpleural emphysema, and multiple purulent abscesses localized within the pulmonary lobes.

Pulmonary tissue samples were fixed in 10% neutral buffered formalin, routinely processed, paraffin‐embedded, sectioned at 4 *μ*m , and stained with hematoxylin and eosin (H&E) for histopathological evaluation at a commercial veterinary pathology laboratory. Histopathological analysis confirmed severe, diffuse neutrophilic bronchopneumonia characterized by dense alveolar and bronchiolar accumulation of viable and degenerated neutrophils, extensive fibrin deposition, necrosis of alveolar walls, widespread capillary congestion, and focal areas of alveolar emphysema. No evidence of neoplastic transformation or foreign material was identified within the examined sections [13, 14].

For microbiological evaluation, lung tissue fragments were collected under strict aseptic surgical techniques using sterile instruments to prevent contamination. Direct postmortem cytological examination using Gram, Ziehl–Neelsen, and rapid panoptic stains demonstrated abundant Gram‐negative bacilli and degenerate neutrophils, without fungal elements or acid‐fast organisms. For microbiological isolation, tissue homogenates were inoculated onto blood agar (5% sheep blood) and tryptic soy agar (TSA) plates and incubated aerobically at 37^∘^C ± 1^∘^C for 24 to 48 h (Figure 5). Morphologically uniform colonies were subcultured onto MacConkey agar to confirm lactose fermentation and purity prior to species identification using the Enterokit B biochemical profile system. Fungal cultures inoculated on Sabouraud dextrose agar yielded no growth after extended incubation [15].

**Figure 5:**
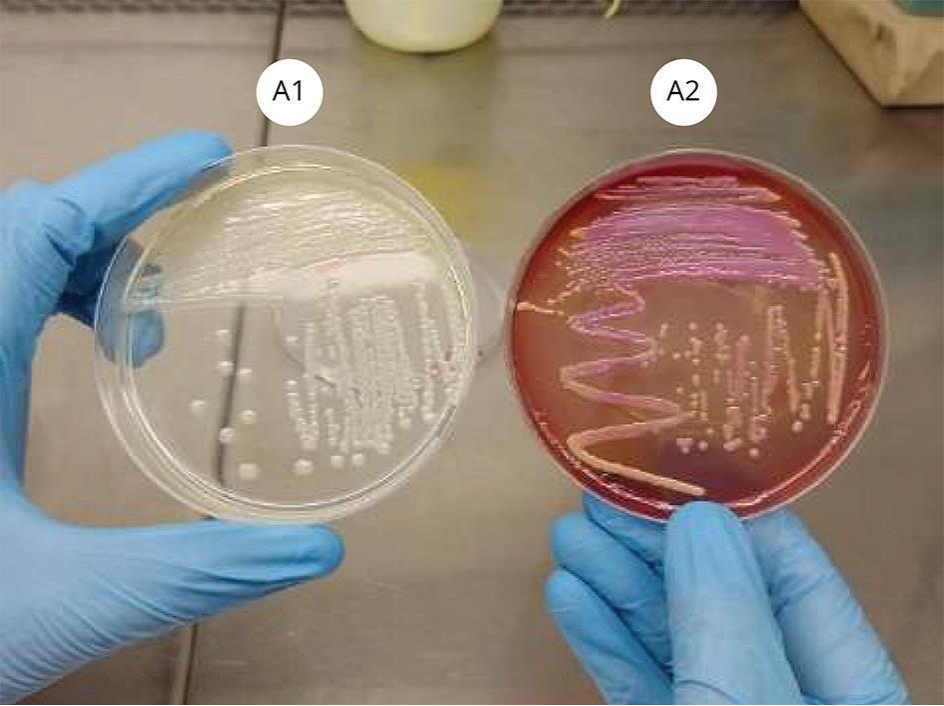
Colony morphology of *Klebsiella pneumoniae* isolated from lung tissue of a pet rat (*Rattus norvegicus*). Pure bacterial growth of *Klebsiella pneumoniae* on (A1) Tryptic Soy Agar and (A2) blood agar demonstrating characteristic mucoid colony morphology following postmortem isolation.

Antimicrobial susceptibility testing was performed using the standardized disk diffusion technique, and interpretive criteria were applied according to the Brazilian Committee on Antimicrobial Susceptibility Testing (BrCAST) guidelines [16]. MDR was operationally defined as nonsusceptibility (resistant or intermediate) to at least one agent in three or more antimicrobial categories, in accordance with international consensus criteria [8]. The isolate was resistant to doxycycline, chloramphenicol, enrofloxacin, marbofloxacin, and norfloxacin; showed intermediate susceptibility to cefazolin, streptomycin, gentamicin, and trimethoprim–sulfamethoxazole; and was susceptible to amikacin, aztreonam, ceftriaxone, cefotaxime, ceftazidime, and cefepime [8, 16]. The full antimicrobial susceptibility profile is summarized in Table 2.

**Table 2:** Antimicrobial susceptibility profile of *Klebsiella pneumoniae* isolated from the lung tissue of a pet rat (*Rattus norvegicus*).

Antibiotics	Susceptibility test result
*β*‐lactams–cephalosporins	—
Cefazolin	Intermediate
Ceftriaxone	Susceptible
Cefotaxime	Susceptible
Ceftazidime	Susceptible
Cefepime	Susceptible
Monobactams	—
Aztreonam	Susceptible
Aminoglycosides	—
Amikacin	Susceptible
Streptomycin	Intermediate
Gentamicin	Intermediate
Tetracyclines	—
Doxycycline	Resistant
Phenicols	—
Chloramphenicol	Resistant
Sulfonamides	—
Sulfa + trimethoprim	Intermediate
Fluoroquinolones	—
Enrofloxacin	Resistant
Marbofloxacin	Resistant
Norfloxacin	Resistant

## 3. Discussion

Respiratory tract infections are among the primary clinical presentations in companion rodent medicine, with environmental management, high ambient ammonia levels, dusty substrate, poor ventilation, and sudden temperature fluctuations representing key predisposing factors [10]. The etiologic spectrum of rodent respiratory disease commonly includes *Mycoplasma pulmonis*, *Streptococcus pneumoniae*, *Pasteurella pneumotropica*, and *Pseudomonas aeruginosa*, which often act as primary or opportunistic pathogens when host mucosal defenses are compromised [10, 17, 18]. Although *M. pulmonis* remains the predominant pathogen associated with chronic murine respiratory disease, the recovery of a MDR *K. pneumoniae* in pure culture highlights the potential for opportunistic enterobacteria to complicate chronic respiratory syndromes in pet rats [1, 3, 10, 17].

*K. pneumoniae* is an opportunistic Gram‐negative pathogen capable of producing severe suppurative pulmonary lesions, largely mediated by capsular polysaccharides (K antigens) and lipopolysaccharides (LPS) that impair phagocytosis and complement‐mediated clearance [4, 19]. In the present case, the isolate exhibited resistance to doxycycline and fluoroquinolones, which provides a plausible explanation for the failure of initial empirical antimicrobial regimens [1, 3, 8, 16]. However, clinical failure in advanced pulmonary disease cannot be attributed solely to AMR [4, 8] . The presence of extensive, encapsulated pulmonary abscesses severely restricts drug penetration into the lesion core, thereby compromising therapeutic efficacy even when agents display nominal in vitro activity [13].

The administration of anti‐inflammatory therapies during management requires critical evaluation. Systemic prednisolone was initially instituted to mitigate severe acute airway inflammation and respiratory distress. Although short‐term anti‐inflammatory therapy can provide symptomatic relief in obstructive respiratory disease, prolonged or repeated corticosteroid administration, including subsequent inhaled formulations, presents a potential risk of localized or systemic immune suppression, which may have facilitated parenchymal bacterial multiplication and abscess maturation. Furthermore, empirical administration of itraconazole was initiated on D32 because of persistent, nonresponsive consolidation and suspicion of secondary fungal infection; however, the absence of antemortem fungal testing and the subsequent negative postmortem fungal culture demonstrate the limitations of empirical antifungal protocols in small exotic mammal practice [1, 11, 13].

Although the recovery of *K. pneumoniae* in pure culture from multiple internal lung sites strongly supports its involvement in the fatal bronchopneumonia, definitive exclusive causation must be interpreted cautiously. Previous exposure to broad‐spectrum antimicrobials may have suppressed copathogens, such as *Mycoplasma* spp. or fastidious anaerobes, thereby favoring the selective recovery of a single resistant strain. Additionally, because no molecular or viral testing was performed, a primary viral or mycoplasmal insult initiating secondary enterobacterial colonization cannot be entirely excluded [1, 11, 17].

From a One Health perspective, the identification of MDR enterobacteria in companion rodents is of clinical interest because of the close physical contact and shared living environment between pet owners and exotic species [1, 3, 5, 17] . However, because no environmental sampling, feed or bedding testing, or owner screening was conducted, no direct cross‐transmission event, reverse zoonosis, or specific environmental source can be confirmed. Consequently, companion rodents in this context are best conceptualized as potential sentinels or indirect indicators of resistance determinants circulating at the human–animal–environment interface rather than proven reservoirs [3, 9, 10, 20].

### 3.1. Study Limitations

This study has several important limitations that must be acknowledged. First, species identification was performed using biochemical profiling rather than MALDI‐TOF mass spectrometry, 16S rRNA sequencing, or whole‐genome sequencing, which limits definitive discrimination within the *K. pneumoniae* species complex. Second, molecular characterization of resistance genes (e.g., ESBL or plasmid‐mediated quinolone resistance determinants) and broth microdilution for minimum inhibitory concentration (MIC) determination were not available [16]. Third, representative histopathological photomicrographs were not provided in the original veterinary pathology laboratory report; nevertheless, diagnostic confirmation of severe neutrophilic bronchopneumonia was formally established by a certified veterinary pathologist through routine H&E analysis. Fourth, no antemortem microbiological sampling (such as tracheal wash or deep choanal swabbing) was performed during the 32‐day clinical course. Finally, the single‐case design and the absence of epidemiological screening of household contacts or cage mates restrict broader generalizations regarding transmission dynamics or true population prevalence.

## 4. Conclusion

This study documents a case of fatal suppurative bronchopneumonia associated with a MDR *K. pneumoniae* isolate in a pet rat (*R. norvegicus*). The observed resistance to doxycycline and fluoroquinolones offers a plausible explanation for the failure of empirical therapies, although advanced pulmonary abscessation and the lack of antemortem microbiological diagnostics also significantly influenced the clinical outcome. Microbiological investigation and antimicrobial susceptibility testing should be considered early in persistent, progressive, or treatment‐refractory respiratory diseases in companion rodents whenever clinically feasible. Because molecular characterization, environmental sampling, and owner screening were not performed, the specific resistance determinants, isolate source, and cross‐species transmission dynamics remain unconfirmed, highlighting companion rodents as potential sentinels for AMR surveillance at the human–animal–environment interface.

## Funding

No funding was received for this manuscript.

## Ethics Statement

All clinical and postmortem procedures complied with national animal welfare legislation (Brazilian Federal Law 11.794/2008) and the *AVMA Guidelines for the Euthanasia of Animals (2020)* [12]. As this study describes nonexperimental, routine clinical diagnostic, and postmortem procedures performed on a client‐owned animal, formal institutional animal care and use committee review was waived in accordance with institutional regulatory guidelines.

## Consent

Written informed consent was obtained from the owner for diagnostic evaluations, therapeutic management, humane euthanasia, postmortem examination, and academic publication of clinical data and figures.

## Conflicts of Interest

The authors declare no conflict of interest.

## Data Availability

The data that support the findings of this study are available on request from the corresponding author. The data are not publicly available due to privacy or ethical restrictions.
